# Global prevalence of adenomyosis and endometriosis: a systematic review and meta-analysis

**DOI:** 10.1186/s12958-025-01483-z

**Published:** 2025-11-19

**Authors:** Mei-Huan Wang, Jia-Hui Chen, Xin-Yu Qi, Zhi-Xun Li, Ying Huang

**Affiliations:** Department of Ultrasound, Shengjing Hospital of China Medical University, Shenyang, Liaoning China

**Keywords:** Adenomyosis, Endometriosis, Dysmenorrhea, Hysterectomy, Epidemiology

## Abstract

**Objective:**

The objective of this meta-analysis is to provide a comprehensive and up-to-date assessment of the prevalence of endometriosis and adenomyosis, addressing the limitations of existing research data and evaluating the quality of the data.

**Methods:**

Adhering to the PRISMA 2020 guidelines, this study systematically searched the Web of Science, PubMed, Embase, and Google Scholar databases from their inception until January 2025 for studies related to adenomyosis and endometriosis. All studies that specifically reported the prevalence of adenomyosis and endometriosis, or provided data enabling calculation of their prevalence, were included. The research examined the occurrence of different subtypes of adenomyosis and endometriosis, as well as frequency in various populations. Furthermore, subgroup analyses were conducted to synthesize findings related to distinct diagnostic criteria, gynecological symptoms, and the prevalence of these two conditions across diverse populations. Meta-analyses were performed using *meta* software packages, and random-effects models with 95% confidence intervals (CI) were employed to determine the combined prevalence of adenomyosis.

**Results:**

A total of 198,925,726 women from 127 studies (59 on adenomyosis and 68 on endometriosis) were included in this meta-analysis. The prevalence of focal and diffuse adenomyosis was found to be 17% (95%CI, 7%-30%) and 15% (95%CI, 9%-23%), respectively. The prevalence of peritoneal endometriosis, ovarian endometriosis, and deep endometriosis was recorded at 6% (95%CI, 1%-15%), 13% (95%CI, 5%-24%), and 10% (95%CI, 2%-24%), respectively. The prevalence of adenomyosis and endometriosis among women experiencing infertility was 31% (95%CI, 10%-58%) and 38% (95%CI, 25%-51%), respectively. Furthermore, among patients experiencing gynecologic symptoms, the pooled of adenomyosis and endometriosis was 41%-49%, and 18%-42%, respectively. The global prevalence of adenomyosis and endometriosis in the general population worldwide is 1% (95%CI, 0%-2%), and 5% (95%CI, 2%-9%), respectively.

**Conclusions:**

In conclusion, endometriosis occurs more frequently than adenomyosis across various populations. Notably, it affects nearly 50% of individuals experiencing gynecological symptoms. This study provides valuables support for public health management and emphasizes the importance of prompt intervention and treatment for related conditions.

**Supplementary Information:**

The online version contains supplementary material available at 10.1186/s12958-025-01483-z.

## Introduction

Adenomyosis and endometriosis are related, chronic, and debilitating disorders characterized by the ectopic presence of endometrial tissue, either within the uterine myometrium or at extrauterine sites such as the peritoneal surfaces and ovaries. Clinically, they manifest through a range of symptoms, including infertility and dysmenorrhea [1, 2]. Although these symptoms are not life-threatening, they significantly increase the challenges faced by women [3, 4]. Therefore, having access to essential epidemiological data is crucial for timely and effective intervention in patients with these conditions. Such data also play a vital role in public health management and decision-making, as well as in investigating of the causes and risk factors associated with these diseases [5]. However, the limited focus on non-cancerous diseases and the heterogeneous nature of adenomyosis and endometriosis have resulted in complex and varied epidemiological data, leading to a lack of comprehensive and consistent conclusions.

The prevalence of endometriosis and adenomyosis varies significantly across different populations, with considerable variation even within the same population. For instance, among patients exhibiting clinical symptoms, the prevalence of endometriosis ranges from 25% to 66% [6, 7], while adenomyosis prevalence varies from 12% to 55% [6, 8]. Additionally, the prevalence of endometriosis in infertile women ranges from 22% to 91% [9, 10], whereas adenomyosis prevalence ranges from 4% to 89% [11, 12]. Given this complex data landscape, it is essential to utilize meta-analytic research to consolidate and synthesize the relevant findings.

Recent meta-analyses have reported that the prevalence of adenomyosis is 10% among women with infertility and 22.6% in cases of endometrial cancer [13, 14]. In contrast, meta-analyses and narrative reviews on the prevalence of endometriosis have primarily included studies conducted before 2017 and 2021, respectively [15, 16]. However, the existing pooled analyses of adenomyosis are limited, and those for endometriosis are not sufficiently up to date. Therefore, there is a clear need for a comprehensive and current meta-analysis to consolidate epidemiological data on these conditions.

This study aims to provide a comprehensive epidemiological profile of adenomyosis and endometriosis through a systematic review and meta-analysis. ​The primary objectives are:​​ [1] ​to estimate the pooled prevalence​ of each condition; [2] to examine prevalence within specific patient subgroups, such as individuals experiencing infertility or those presenting with gynecological symptoms; and [3] to evaluate the influence of various diagnostic methodologies, including imaging and histological techniques, on prevalence estimates. Furthermore, the study will assess heterogeneity among the included studies and address the potential for publication bias.

## Methods

### Data sources and search strategy

The review was registered prospectively with PROSPERO (CRD42025642611). The systematic review was reported in line with the Preferred Reporting Items for Systematic Reviews and Meta-Analyses (PRISMA) 2020 checklist [17]. A thorough literature review was conducted using the Web of Science, PubMed, Embase, and Google Scholar databases, covering the period from their inception until January 10, 2025. The search was performed using a combination of keywords and terms for adenomyosis, endometriosis (‘adenomyoma’ OR ‘adenomyosis uteri’ OR ‘endometriosis’ OR ‘endometrial adenoma’) and prevalence (‘occurrence’ OR ‘incidence’ OR ‘epidemiology’ OR ‘frequency’), as the search details for the different databases were shown in Table S1.

The following inclusion criteria were applied: [1] Clearly define the diagnosis of endometriosis or adenomyosis [2], The prevalence of endometriosis or adenomyosis is reported or available for calculation [3], Original research and full text accessible.

The following exclusion criteria were used: [1] The prevalence of adenomyosis was not mentioned in the publication, and no data to calculate the prevalence is included [2]. Case reports, reviews, abstracts, or comments [3]. Duplicate articles and research with no full-text available. The research language was not restricted, and the reference lists of all studies considered were reviewed to make sure that all potentially relevant studies were taken into account.

### Study selection

Two reviewers independently evaluated the retrieved studies independently based on predefined inclusion and exclusion criteria. Firstly, duplicate research in various databases was deleted based on the search method. Second, unnecessary articles were eliminated by reading the article title and abstract. Finally, articles with insufficient data and duplicate data were excluded after reading the full text. The differences between the two reviewers are addressed through the involvement of a third party for resolution.

### Data extraction

Study data were extracted following standard procedures, including the first author, publication year, country, study design, age, population characteristics, survey period, inclusion criteria and exclusion criteria, diagnostic methods, number of patients, total sample size, and diagnostic criteria for diseases. Data on adenomyosis and endometriosis were extracted and analyzed separately and independently throughout the review process. The studies were categorized by publication year into three intervals (prior to 2010, 2010–2017, and 2017–2024) to examine temporal trends. This stratification was intentionally aligned with significant advancements in diagnostic imaging criteria and clinical practice guidelines, such as the MUSA consensus and ESHRE guidelines, which likely influenced the identification and reported prevalence rates of adenomyosis and endometriosis.

### Assessment of risk of bias

The evaluation of research quality was conducted using the Joanna Briggs Institute (JBI) checklist, a standardized and widely recognized tool for assessing the methodological rigor of epidemiological studies [18]. The JBI checklist consists of nine items, each rated as ‘Yes’ (scored 1) or ‘No,’ ‘Unclear,’ and ‘Not Applicable’ (each scored 0). The total score ranges from 0 to 9, with higher scores indicating better quality. Additionally, a sensitivity analysis was performed to assess the robustness of the findings by excluding studies rated as low quality (score ≤ 4) according to the JBI assessment tool.

### Data synthesis

Data analysis was performed using software R (version 4.2.0). The raw prevalence proportions were transformed using the Freeman-Tukey double arcsine transformation to stabilize their variances prior to pooling. This transformation was specifically chosen because it provides robust variance stabilization across the entire range of possible values (0% to 100%) and allows for the inclusion of extreme proportions without the need for arbitrary continuity corrections, which is a limitation of alternative methods like the logit transform [19]. Meta-analyses were performed using the *meta* and *metafor* software packages to establish the pooled prevalence of adenomyosis and endometriosis [20]. Heterogeneity was assessed using the I² statistic and the tau² (Tau-squared) statistic. In line with Cochrane guidelines, I^2^ values greater than 50% were considered to indicate substantial heterogeneity. The tau² statistic was used to estimate the actual amount of variance between studies [21]. A random-effects model was employed for all meta-analyses, as we anticipated clinical and methodological heterogeneity among the included studies [22].

Meta-analytical methods were utilized to examine the prevalence of endometriosis and adenomyosis across diverse populations, along with the prevalence of their corresponding subtypes. Subgroup analyses were conducted to thoroughly examine the prevalence of both conditions across various populations and clinical contexts. Specifically, the analysis focused on the prevalence of adenomyosis in relation to different diagnostic modalities, hysterectomy, symptomatic women, and parity status. Correspondingly, the prevalence of endometriosis was assessed across diverse populations and diagnostic methods. Furthermore, all meta-analyses were performed ​separately for adenomyosis and endometriosis. Data from studies reporting on both conditions were extracted and analyzed independently for each condition.

Publication bias refers to the tendency for studies with favorable results to be published more frequently than those with negative or null findings [23]. Because observational studies of prevalence do not typically report positive or negative outcomes, we did not conduct publication bias analyses.

## Results

### Study selection

**Fig. 1:**
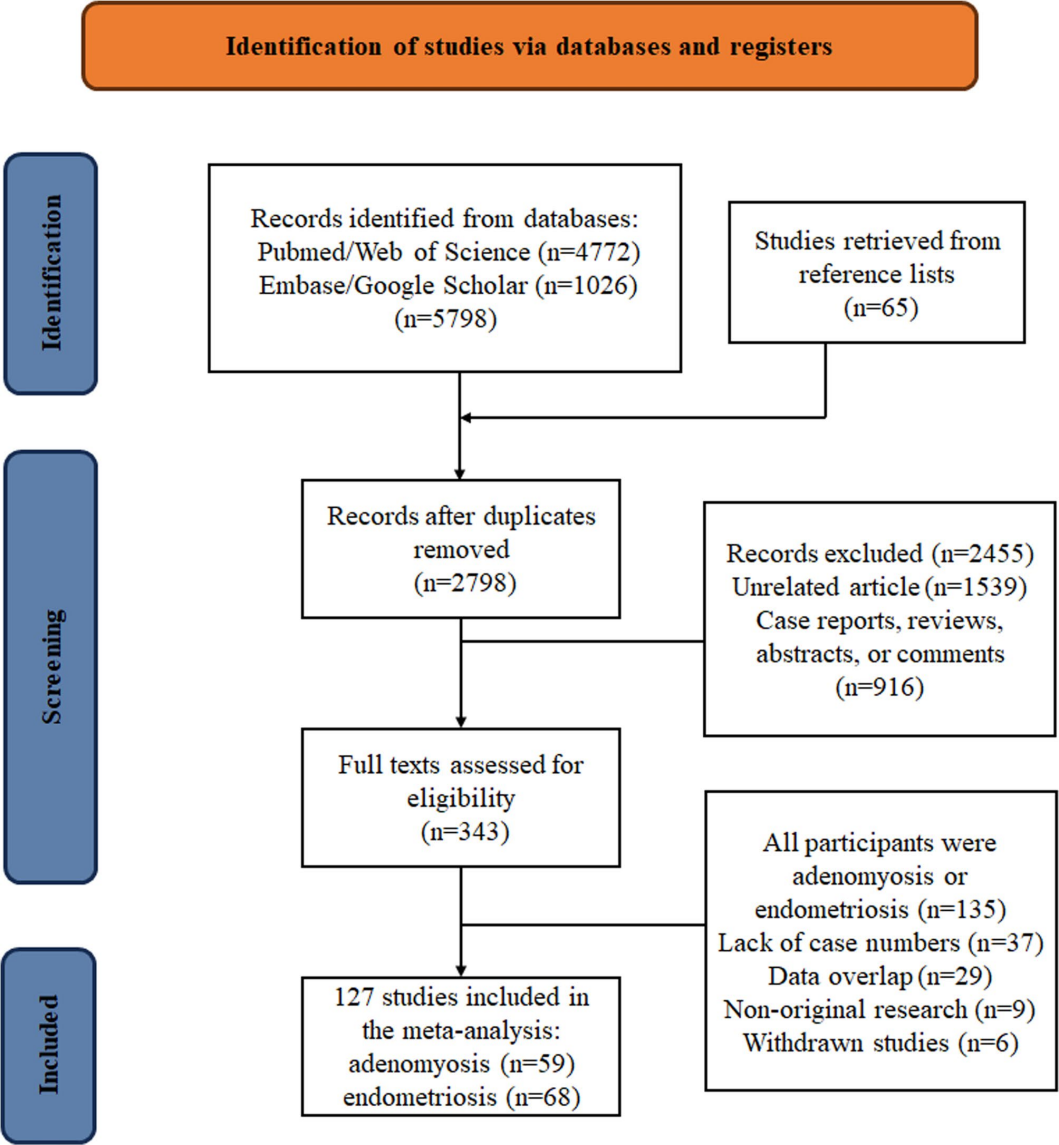
Flowchart of the meta-analysis selection process

Out of the initial 5,789 studies identified, along with 65 additional studies obtained from reference lists, a total of 2,798 studies remained for further analysis after duplicates were removed. Following the exclusion of studies deemed irrelevant or not meeting eligibility criteria, 343 studies were selected for full-text review. Ultimately, 127 studies were included, comprising 59 focused on adenomyosis and 68 on endometriosis (Fig. 1). Table 1 summarizes the general characteristics of the included literature, while Tables S2 and S4 detail specific features and diagnostic criteria for adenomyosis research. Tables S3 and S5 provide corresponding information related to endometriosis.

**Table 1:** Summary of baseline characteristics of the included studies

	No. studies	Study design	Risk of bias (average score)	Sample size	Diagnostic modality
Adenomyosis	59				
Published before 2010	14	Prospective, Retrospective, Cross-sectional	7.1	128,248	Histologic pathology, MRI
Published between 2010–2017	19	Prospective, Retrospective, Cross-sectional	6.8	815,228	MRI, Histologic pathology, TVS
Published between 2017–2024	26	Prospective, Retrospective, Cross-sectional	7.2	345,005	MRI, Histologic pathology, TVS, Gynaecological examination
Endometriosis	68				
Published before 2010	17	Prospective, Retrospective, Cross-sectional, Cae-control	7.0	191,396,578	Laparoscopy, Medical interview, physical, clinical, and ultrasound examination, Histologic pathology, MRI, Self-reported
Published between 2010–2017	17	Prospective, Cross-sectional, Retrospective,	7.2	15,170,984	Self-reported, Laparoscopy, Hospitalization diagnosis, Laparoscopy, MRI, Histologic pathology
Published between 2017–2024	34	Retrospective, Cross-sectional, Prospective,	7.5	176,224,303	Self-reported, Surgery, US, Histologic pathology, Laparoscopy, MRI

### Study characteristics

The majority of studies reporting the prevalence of adenomyosis were from Europe (54.2%, 32/59), followed by Asia (23.7%, 14/59). A total of 34 studies employed histological analysis as the diagnostic standard, 9 studies utilized MRI, 14 studies relied on ultrasound examination, and 2 studies applied a combination of diagnostic criteria. The prevalence of adenomyosis in women undergoing hysterectomy was reported in 30 studies, and the prevalence of adenomyosis in women with endometriosis was recorded in 14 studies. Adenomyosis was seen in symptomatic populations with AUB (including menorrhagia and menometrorrhagia), pelvic pain, and dysmenorrhea, according to sixteen, ten, and twelve studies, respectively.

In the research concerning endometriosis, most of the studies originated from Europe, accounting for 47.06% (32 out of 68), while Asia contributed 16.18% (11 out of 68). Endometriosis was diagnosed most commonly with laparoscopy (38.24%, 26/68), followed by histology and imaging (MRI and US) in 11 and 8 studies, respectively. Furthermore, a total of 25 extensive studies have documented the prevalence of endometriosis within the general population, whereas 12 studies have focused specifically on its prevalence among women experiencing infertility.

### Risk of bias of included studies

The quality of the studies incorporated in this analysis was evaluated using the JBI checklist, with the results summarized in Table S6. Among the 127 studies reviewed, only two focused on endometriosis were classified as low quality, each scoring exactly 4 points. The quality of the studies that reported the prevalence of adenomyosis and endometriosis was found to be similar, with mean scores of 7.0 for adenomyosis and 7.4 for endometriosis.

### Synthesis of results

**Fig. 2:**
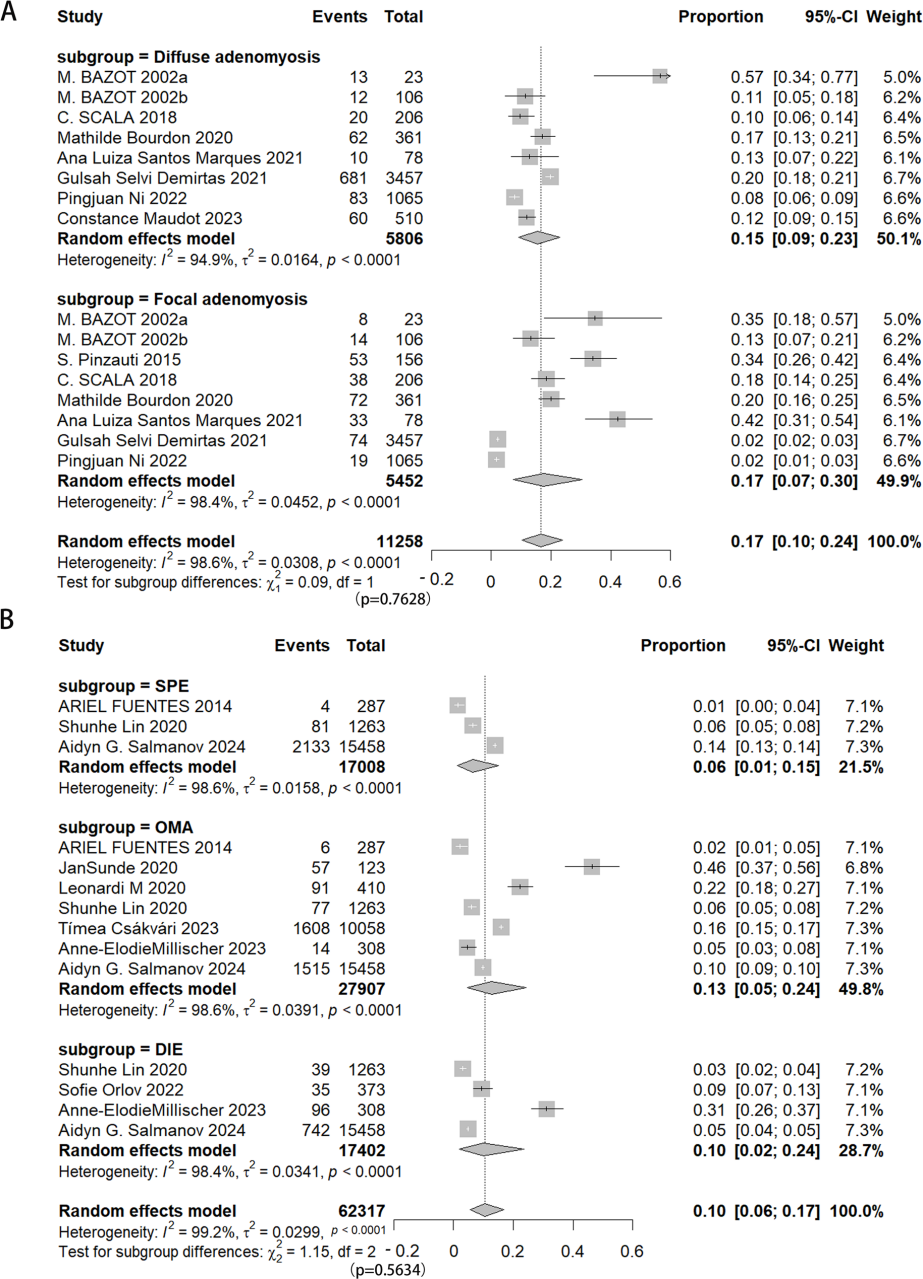
Forest plot showing the prevalence of adenomyosis (**A**) and endometriosis (**B**) subtypes

Eight studies indicated that the prevalence of focal and diffuse adenomyosis was comparable, with rates of 17% and 15%, respectively (Fig. 2A). Additionally, eight studies examined the prevalence of superficial peritoneal endometriosis (SPE), ovarian endometrioma (OMA), and deep infiltrating endometriosis (DIE), revealing a slightly higher occurrence of endometriosis in the ovaries at 13%, compared to 6% in the SPE and 10% in DIE (Fig. 2B). Significant heterogeneity was observed across the studies pertaining to these subtypes (I^2^ = 98%−99%, tau^2^ = 0.0158–0.0391).

**Fig. 3:**
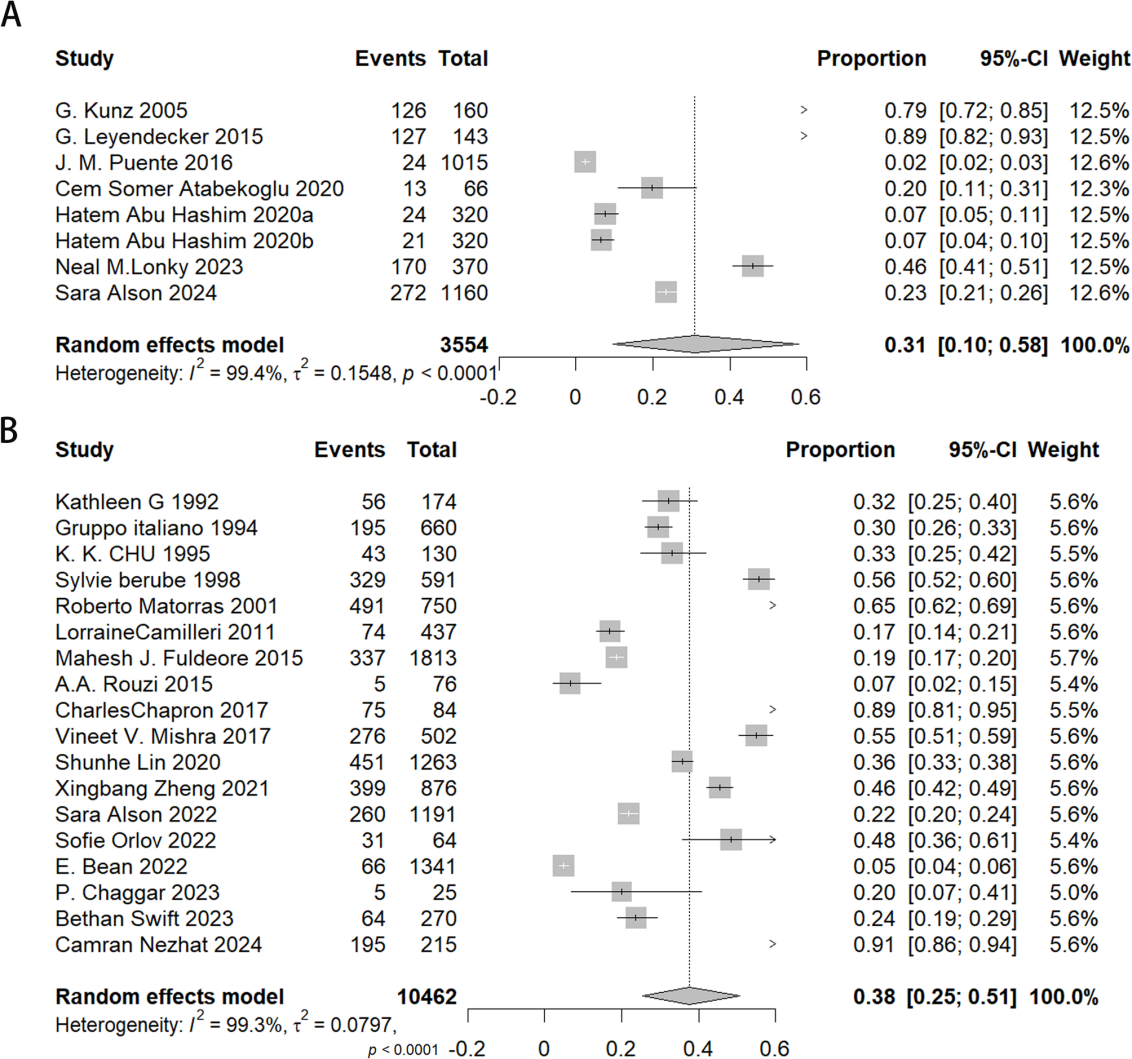
Forest plot showing the prevalence of adenomyosis (**A**) and endometriosis (**B**) in women with subfertility

This research investigated the prevalence of adenomyosis and endometriosis among individuals experiencing infertility, with the results illustrated in Fig. 3. The prevalence of adenomyosis was derived from eight estimates across seven studies, yielding a combined prevalence rate of 31% (95% CI, 10%−58%, I^2^ = 99%, tau^2^ = 0. 1548). Similarly, the prevalence of endometriosis was derived from eighteen studies, yielding a combined prevalence rate of 38% (95% CI, 25%−51%, I^2^ = 99.3%, tau^2^ = 0.0797).

Subgroup analyses were conducted in this meta-analysis to examine the prevalence of adenomyosis across various populations and conditions (Table 2). When it comes to diagnostic methods, histopathology (35.1%; 95%CI, 30.9%−39.4%) and MRI (35.0%; 95%CI, 22.6%−48.4%) reveal a greater prevalence of adenomyosis compared to ultrasound (30.7%; 95%CI, 25.2%−48.4%). Furthermore, the prevalence of adenomyosis was greater among individuals who have a hysterectomy for benign conditions (42.3%; 95%CI, 35.8%−48.9%) than among those who have the procedure for malignant conditions (31.9%; 95%CI, 15.1%−51.4%).When comparing various populations, it was found that the occurrence of adenomyosis among women who had given birth was greater than that in nonpregnant women. Additionally, the prevalence of adenomyosis in women experiencing gynecological symptoms was comparable to that in those with endometriosis and exceeded that in women with uterine fibroids. Ultimately, this prevalence is 1% within the general population, which serves as the most accurate reflection of epidemiological census data.

**Table 2:** Epidemiological subgroup analysis of adenomyosis and endometriosis

	Studies (*n*)	Prevalence (95% CI)	Tau^2^	I^2^	*P*
Adenomyosis					
Diagnostic modality					
US	16	0.31 (0.25–0.37)	0.0355	94%	< 0.01
MUSA	9	0.29 (0.14–0.48)	0.0852	99%	< 0.01
MRI	9	0.35 (0.23–0.48)	0.0794	98%	< 0.01
Histologic pathology	34	0.35 (0.32–0.39)	0.0435	100%	< 0.0001
Gravidity					
Parous	7	0.38 (0.29–0.48)	0.0129	93%	< 0.01
Nulliparous	7	0.28 (0.18–0.39)	0.0225	92%	< 0.01
Symptomatic women					
AUB	13	0.42 (0.29–0.57)	0.0636	98%	< 0.01
Pelvic pain	12	0.49 (0.42–0.56)	0.0119	81%	< 0.01
Dyspareunia	7	0.46 (0.34–0.57)	0.0186	86%	< 0.01
Dysmenorrhea	12	0.41 (0.31–0.51)	0.0282	95%	< 0.01
Causes of hysterectomy					
Benign disease	22	0.42 (0.36–0.49)	0.0227	93%	< 0.01
Malignant disease	2	0.32 (0.15–0.51)	0.0158	77%	0.04
Benign and malignant diseases	10	0.26 (0.19–0.34)	0.0184	95%	< 0.01
Different population					
Endometriosis	14	0.42 (0.30–0.54)	0.0415	96%	< 0.01
Myoma	11	0.37 (0.24–0.50)	0.0459	94%	< 0.01
General population	3	0.01 (0.00–0.02)	0.0028	100%	< 0.0001
Endometriosis					
Stages					
I	7	0.17 (0.10–0.26)	0.0193	98%	< 0.01
II	7	0.05 (0.04–0.07)	0.0028	86%	< 0.01
III	7	0.04 (0.03–0.06)	0.0008	65%	< 0.01
IV	7	0.04 (0.02–0.06)	0.0047	91%	< 0.01
Diagnostic modality					
Laparoscopy	17	0.31 (0.23–0.41)	0.0444	98%	< 0.01
Histopathology	10	0.17 (0.05–0.34)	0.1005	100%	< 0.0001
Laparoscopy or Histopathology	9	0.20 (0.05–0.42)	0.1363	100%	< 0.0001
Self-reported	10	0.04 (0.03–0.06)	0.0063	100%	< 0.0001
US	5	0.18 (0.10–0.27)	0.0157	98%	< 0.01
MRI	3	0.25 (0.04–0.56)	0.0818	100%	< 0.01
Other	14	0.07 (0.01–0.19)	0.1310	100%	< 0.0001
Symptomatic women					
Dysmenorrhea	9	0.42 (0.20–0.64)	0.1193	100%	< 0.0001
Pelvic pain	12	0.27 (0.17–0.39)	0.0447	100%	< 0.0001
Dyspareunia	8	0.18 (0.06–0.35)	0.0564	99%	< 0.01
Different population					
General population	25	0.05 (0.02–0.09)	0.0552	100%	< 0.0001
Parous	11	0.26 (0.14–0.39)	0.0567	98%	< 0.01
Nulliparous	23	0.34 (0.25–0.44)	0.0577	99%	< 0.01

Abbreviations: *AUB* Abnormal uterine bleeding, *MUSA* Morphological Uterus Sonographic Assessment, *US* Ultrasound, *MRI* Magnetic Resonance Imaging

Subgroup analyses of endometriosis examined the disease’s prevalence in various diagnostic modalities and populations. In terms of diagnostic modality, laparoscopically diagnosed endometriosis had the highest prevalence (31%; 95%CI, 23%−41%), followed by histology and imaging (MRI and US) reported prevalence, and the lowest prevalence self-reported via questionnaires (4%; 95%CI, 3%−6%). The frequency of endometriosis in the population with gynaecological symptoms is equivalent to that of adenomyosis (1%), although endometriosis is more common in the general population than in adenomyosis (5%; 95%CI, 2%−9%). Furthermore, the prevalence of endometriosis is higher among nulliparous women (34%; 95%CI, 25%−44%) compared to parous women (26%; 95%CI, 14%−39%).

Table S7 presents the results of a meta-regression analysis based on subgroups. Most subgroups showed no statistically significant differences in the prevalence of adenomyosis (*P* > 0.05). However, there were statistically significant differences among patients with adenomyosis who underwent hysterectomy for various reasons (*P* = 0.0025) and among patients with endometriosis across different populations (*P* < 0.0001).

### Sensitivity analysis

Following the exclusion of two studies deemed to be of low quality, the prevalence within the endometriosis subgroup remained largely unchanged, as shown in Table S8. Compared to the findings in Table 2, excluding low-quality studies did not significantly alter the prevalence rates within the respective subgroups.

## Comment

### Principal findings

This research analyzed extensive epidemiological data on adenomyosis and endometriosis from 127 studies involving 198,925,726 female patients. The prevalence of focal and diffuse adenomyosis is 17% and 15%, respectively, while the prevalence of SPE, OMA, and DIE is 6%, 13%, and 10%, respectively. Among the infertile population, the prevalence of adenomyosis and endometriosis is 31% and 38%, respectively, compared to 1% and 5% in the general global population. Furthermore, in patients presenting with gynecologic symptoms, the prevalence of adenomyosis ranges from 41% to 49%, whereas endometriosis occurs in 18% to 42% of cases.

### Comparison with existing literature

Infertility is a significant consequence of both adenomyosis and endometriosis. In this study, the prevalence of infertility associated with adenomyosis and endometriosis was reported to be similar, at 31% and 38%, respectively. These findings align with the results of two previous studies [15, 24]. J.M. Puente et al. found that adenomyosis was present in 38.2% of individuals with recurrent miscarriage and 34.7% of those with failed assisted reproductive technology (ART) treatments [24]. A systematic review conducted by Yousef Moradi et al. reported that the prevalence of endometriosis among infertile patients was 31% [15]. Moreover, various subtypes of adenomyosis and endometriosis may have differential impacts on maternal outcomes. DIE has been identified as a risk factor for spontaneous hemoperitoneum during gestation, whereas diffuse adenomyosis is associated with a threefold increase in the relative risk of miscarriage [3, 25]. Additional research is warranted to investigate the prevalence of distinct adenomyosis and endometriosis subtypes among patients experiencing infertility.

Apart from causing infertility, adenomyosis and endometriosis are commonly associated with various gynecological symptoms, such as dysmenorrhea, pelvic pain, and dyspareunia. Adenomyosis accounts for approximately 41% to 49% of women experiencing these symptoms, consistent with earlier research that identified dysmenorrhea and dysuria as risk factors for the condition [26]. However, the prevalence of endometriosis among patients with pelvic pain was found to be 27%, which is lower than the 42% to 44% reported in previous studies [15, 16]. A key factor contributing to this discrepancy is that this study utilized data from two large population samples from Spain and the United States [27, 28], potentially leading to a lower prevalence compared to smaller samples drawn from gynecological clinics.

It is widely recognized that the risk factors for adenomyosis include growing age, parity, and a history of uterine surgery [29]. Our subgroup analysis found that multiparous women (38%) had a greater prevalence of adenomyosis than nulliparous women (28%), which is consistent with previous study [30]. However, subgroup analyses indicated that nulliparous women have a higher prevalence of endometriosis than multiparous women. This may be related to the greater prevalence of endometriosis found in infertile women, as infertile women are categorized as nulliparous in this study. Consequently, future research should focus on the influence of parity on the pathophysiology of endometriosis and its effects on pregnancy outcomes. Moreover, adenomyosis is found in 42% of individuals with endometriosis and 37% of those with uterine fibroids. This significant overlap supports the hypothesis that adenomyosis and endometriosis may be different manifestations of the same fundamental issue [31]. Additionally, a growing body of research has shown that internal adenomyosis is more commonly associated with uterine fibroids, whereas external adenomyosis is more often linked to endometriosis [32, 33].

### Strengths and limitations

This study provides comprehensive and current epidemiological data on adenomyosis and endometriosis. First, it summarizes the prevalence of focal and diffuse adenomyosis for the first time. Additionally, the prevalence of SPE and OMA and DIE, as well as the prevalence of stage I to IV endometriosis, are summarized in accordance with previous studies. Secondly, this research examines the occurrence of the two diseases in relation to different symptoms and demographic groups. This data is crucial for informing the creation of public health policies and for facilitating early intervention and treatment for those impacted. Our study, however, has some unavoidable drawbacks. First, due to the extensive time period covered by our research (from 1996 to 2024), there may be discrepancies in the diagnostic criteria for adenomyosis and endometriosis, which could lead to variations in prevalence rates. Moreover, the considerable variability arising from the unique features of the studies included should be taken into account, which calls for careful consideration when interpreting the findings. Furthermore, studies presenting extreme prevalence estimates, whether notably high or low, or reporting novel associations, may have a higher likelihood of publication, which could affect the overall pooled estimate. Nonetheless, due to the absence of robust methodologies to accurately assess this bias in prevalence data, the findings of this study should be considered with this potential limitation acknowledged.

## Conclusions and implications

In conclusion, adenomyosis and endometriosis affect approximately 1% and 5% of the global population, respectively. Among patients experiencing infertility, these rates increase to 31% and 38%. In individuals with gynecological symptoms, the prevalence may be even higher, potentially approaching 50%.

## Supplementary Information

Supplementary Material 1.

## Data Availability

The data that support the findings of this study are available from the corresponding author upon reasonable request.
